# Spatiotemporal transcriptome analysis reveals critical roles for mechano-sensing genes at the border zone in remodeling after myocardial infarction

**DOI:** 10.1038/s44161-022-00140-7

**Published:** 2022-11-17

**Authors:** Shintaro Yamada, Toshiyuki Ko, Satoshi Hatsuse, Seitaro Nomura, Bo Zhang, Zhehao Dai, Shunsuke Inoue, Masayuki Kubota, Kosuke Sawami, Takanobu Yamada, Tatsuro Sassa, Mikako Katagiri, Kanna Fujita, Manami Katoh, Masamichi Ito, Mutsuo Harada, Haruhiro Toko, Norifumi Takeda, Hiroyuki Morita, Hiroyuki Aburatani, Issei Komuro

**Affiliations:** 1 Department of Cardiovascular Medicine, Graduate School of Medicine, University of Tokyo, Tokyo, Japan; 2 Genome Science Division, Research Center for Advanced Science and Technologies, University of Tokyo, Tokyo, Japan

**Keywords:** Transcriptomics, Myocardial infarction

## Abstract

The underlying mechanisms of ventricular remodeling after myocardial infarction (MI) remain largely unknown. In this study, we performed an integrative analysis of spatial transcriptomics and single-nucleus RNA sequencing (snRNA-seq) in a murine MI model and found that mechanical stress-response genes are expressed at the border zone and play a critical role in left ventricular remodeling after MI. An integrative analysis of snRNA-seq and spatial transcriptome of the heart tissue after MI identified the unique cluster that appeared at the border zone in an early stage, highly expressing mechano-sensing genes, such as *Csrp3*. AAV9-mediated gene silencing and overexpression of *Csrp3* demonstrated that upregulation of *Csrp3* plays critical roles in preventing cardiac remodeling after MI by regulation of genes associated with mechano-sensing. Overall, our study not only provides an insight into spatiotemporal molecular changes after MI but also highlights that the mechano-sensing genes at the border zone act as adaptive regulators of left ventricular remodeling.

## Abstract (web summary)

Komuro et al. performed an integrative analysis of single-nucleus RNA sequencing and spatial transcriptome analysis of the injured heart to map cellular and molecular changes across topographical domains relative to the site of injury, and they identify that mechano-sensing genes at the border zone act as adaptive regulators of left ventricular remodeling.

## Main

Despite decades of intensive research and therapeutic developments, ischemic heart disease remains a leading cause of death worldwide. Myocardial infarction (MI) causes various cardiac complications, such as arrhythmia, valvular disease and heart failure, and heart failure is becoming a more serious problem in many countries^1^. MI induces changes in left ventricular (LV) size, shape and function that are considered to be LV remodeling processes and known to initiate the development of heart failure^2–4^. Therefore, it is paramount to clarify the molecular mechanisms underlying LV remodeling after MI to inhibit the development of heart failure and improve the prognosis of patients with MI^5–8^. However, because cellular and molecular behaviors change spatially and temporally after MI, it is difficult to precisely understand the molecular mechanisms associated with LV remodeling.

The infarcted heart is usually roughly divided into three zones: an infarct zone (IZ), an ischemic border zone (BZ, defined as the sectors adjacent to the IZ) and a remote zone (RZ)^9,10^. It was reported that distinct cell type of hypo-contractile myocardium seen in the BZ would extend to involve contiguous normal myocardium during post-infarction remodeling and ultimately induce infarction expansion^9^. The BZ also remodels electrophysiologically and can represent the origin of ventricular arrhythmia^11^. There are transcriptional differences between myocardium proximal and distal to the IZ^10,12–15^. Cardiomyocytes in the BZ undergo a profound transcriptional and epigenetic reprogramming, switching from a MEF2-responsive to an AP-1-responsive gene program^10^. Expression of some long non-coding RNAs (lncRNAs) was upregulated in the BZ compared to the RZ and might be involved in maladaptive remodeling, cardiac function and possibly cardiac regeneration^12^. These previous studies have tried to clarify transcriptomic changes in each region after MI, but they examined gene expression profiles using mRNA isolated from bulk samples, and, thus, it was difficult to dissect the molecular mechanisms underlying LV remodeling at the single-cell level. On the other hand, singe-cell RNA sequencing (scRNA-seq) analyses of MI have revealed dynamic transcriptomic changes at the single-cell level after MI in cardiomyocytes^2,16^, cardiac fibroblasts^15,16^ and endothelial cells^17,18^. In these studies, however, in spite of the importance of spatial information after MI, they did not include precise spatial information. In the present study, we identified and characterized the distinct transcriptional properties associated with each region of infarcted hearts using integrative analysis of single-nucleus RNA sequencing (snRNA-seq) and spatial transcriptomics. We unveiled a molecular adaptation of cardiomyocytes in the BZ characterized by transcriptional activation of mechano-sensing genes, including *Csrp3* (also known as *MLP* (muscle LIM protein)).

## Results

### snRNA-seq reveals the spatiotemporally distinct clusters

**Fig. 1:**
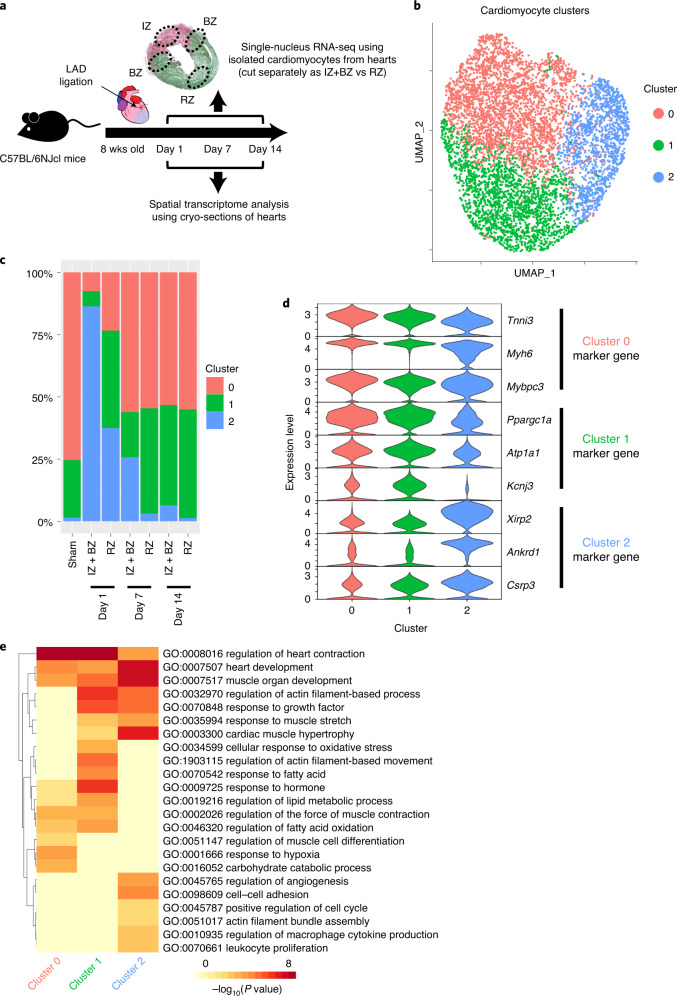
snRNA-seq identifies spatiotemporally distinct cell clusters after MI. **a**, Experimental scheme of single-nucleus and spatial transcriptomic analysis after MI. snRNA-seq was performed on sham, *n* = 2; IZ + BZ on pMI day 1, *n* = 2; RZ on pMI day 1, *n* = 2; IZ + BZ on pMI day 7, *n* = 2; RZ in pMI day 7, *n* = 2; IZ + BZ on pMI day 14, *n* = 2; and RZ on pMI day 14, *n* = 2. Spatial transcriptome was performed on sham, *n* = 1; pMI day 1, *n* = 3; pMI day 7, *n* = 3; and pMI day 14, *n* = 3. **b**, UMAP plot of cardiomyocyte subsets from snRNA-seq. All cardiomyocytes were classified into three clusters (Clusters 0–2). Each nucleus (dot) was colored by clusters. Total cardiomyocytes, *n* = 6,813; sham, *n* = 417; IZ + BZ on pMI day 1, *n* = 1,070; RZ on pMI day 1, *n* = 521; IZ + BZ on pMI day 7, *n* = 894; RZ on pMI day 7, *n* = 1,281; IZ + BZ on pMI day 14, *n* = 1,594; and RZ on pMI day 14, *n* = 1,036. **c**, Bar plot showing the distribution of clusters at each timepoint and region. **d**, Violin plot showing gene expression levels of representative DEGs in each cluster. **e**, Heat map showing the results of GO enrichment analysis for each cluster. Enrichment *P* values were generated by Metascape using cumulative hypergeometric distributions. LAD, left anterior descending. Source data

**Extended Data Fig. 1:**
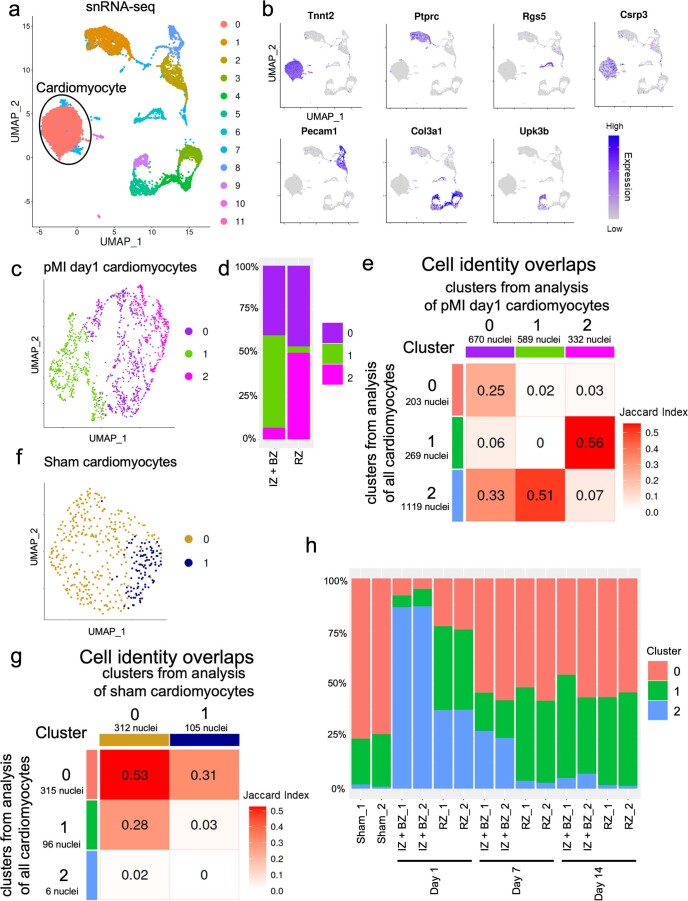
Single-nucleus RNA-seq of the hearts after myocardial infarction. **a**, UMAP plot of all nuclei from single-nucleus RNA-seq of the hearts after myocardial infarction (sham, n = 2; IZ + BZ on pMI day 1, n = 2; RZ on pMI day 1, n = 2; IZ + BZ on pMI day 7, n = 2; RZ in pMI day 7, n = 2; IZ + BZ on pMI day 14, n = 2; RZ on pMI day 14, n = 2). All nuclei were classified into twelve clusters (cluster 0-11). Each nucleus (dot) was colored by clusters. Cardiomyocytes belong to cluster 0 and cluster 1. Total nuclei, n = 12787; sham, n = 595; IZ + BZ on pMI day 1, n = 2474; RZ on pMI day 1, n = 682; IZ + BZ on pMI day 7, n = 3004; RZ on pMI day 7, n = 1793; IZ + BZ on pMI day 14, n = 2895; RZ on pMI day 14, n = 1344. **b**, Visualization of expression of known cell markers used for cell-type annotation on UMAP plot. *Tnnt2*, cardiomyocyte; *Ptprc*, immune cell; *Rgs5*, pericyte, and smooth muscle cell; *Pecam1*, endothelial cell; *Col3a1*, fibroblast; *Upk3b*, epicardial cell. **c**, UMAP plot based on analysis of cardiomyocytes in the pMI day 1 (IZ + BZ on pMI day 1, n = 2; RZ on pMI day 1, n = 2) condition. Cardiomyocytes on pMI day 1 were classified into three clusters (cluster 0-2). Each nucleus (dot) was colored by clusters. IZ + BZ on pMI day 1, n = 1070; RZ on pMI day 1, n = 521. **d**, Bar plot showing the distribution of pMI day 1 clusters at each time point and region. **e**, Heatmap showing the significance of cell identity overlap between clusters of cardiomyocytes and clusters of pMI day 1 cardiomyocytes analyzed. The table is colored following the Jaccard index. **f**, UMAP plot based on the analysis of cardiomyocytes in the sham (n = 2) condition. Cardiomyocytes in the sham condition were classified into two clusters (clusters 0-1). Each nucleus (dot) was colored by clusters (n = 417). **g**, Heatmap showing the significance of cell identity overlap between clusters including all cardiomyocytes and clusters of sham cardiomyocytes. The table is colored following the Jaccard index. **h**, Bar plot showing the distribution of clusters of each sample.

We generated a murine MI model by ligating the left anterior descending artery. We separately isolated cardiomyocytes from the IZ + BZ (area including the IZ and 2 mm of its lateral margin) and the RZ (area other than IZ + BZ) and performed time-dependent snRNA-seq after MI. Furthermore, we performed a spatial transcriptomic analysis of the same time course (Fig. 1a and Supplementary Table 1). We analyzed 12,787 nuclei of cardiac cells (sham, *n* = 2; IZ + BZ on post-MI (pMI) day 1, *n* = 2; RZ on pMI day 1, *n* = 2; IZ + BZ on pMI day 7, *n* = 2; RZ on pMI day 7, *n* = 2; IZ + BZ on pMI day 14, *n* = 2; and RZ on pMI day 14, *n* = 2) and 6,813 nuclei of a cardiomyocyte subpopulation exhibiting high *Tnnt2* expression (Extended Data Fig. 1a,b). Clustering analysis classified cardiomyocytes into three cardiomyocyte clusters (Fig. 1b and Supplementary Table 2). Cluster 0 and Cluster 1 were relatively similar, both occupying the major components of the cardiomyocyte populations in the sham hearts and the pMI day 14 hearts (Fig. 1c,d and Extended Data Fig. 1h). Gene Ontology (GO) enrichment analysis showed that genes associated with lipid metabolic process, such as *Ppargc1a* and *Atp1a1*, were more highly expressed in Cluster 1 than in Cluster 0, whereas genes associated with muscle cell differentiation, such as *Mybpc3*, *Myh6* and *Tnni3*, were more highly expressed in Cluster 0 than in Cluster 1 (Fig. 1d,e). Because most cardiomyocytes in the sham hearts belong to either Cluster 0 or Cluster 1, cardiomyocytes from Cluster 0 and Cluster 1 were thought to be cardiomyocytes under normal conditions, whereas the cardiomyocytes from Cluster 2 were distinct from those belonging to Cluster 0 and Cluster 1 (Fig. 1c). GO enrichment analysis showed that highly expressed genes in Cluster 2, such as *Acta1*, *Csrp3*, *Ankrd1, Flnc* and *Xirp2*, were related to actin filament assembly, angiogenesis, cell–cell adhesion and response to muscle stretch (Fig. 1e). Cluster 2 cardiomyocytes were more prevalent in the IZ + BZ of the acute phase than in the RZ of the chronic phase (Fig. 1c). Most cardiomyocytes within the IZ + BZ on pMI day 1 belonged to Cluster 2.

To evaluate the justification of the clustering, we also performed unsupervised clustering for each condition, such as sham and pMI day 1, separately and compared its result with those of the previous clustering for all conditions. The nuclear clusters from clustering analyses of each condition corresponded to those of the previous clustering (Extended Data Fig. 1c–g). For example, cardiomyocytes from the sham hearts were classified into two clusters, whereas cardiomyocytes from the pMI day 1 hearts were classified into three clusters, which corresponded to the result from the clustering of all conditions. Numerous cardiomyocytes from the IZ + BZ on pMI day 1 were allocated to a distinctive cluster as in the previous clustering (Extended Data Fig. 1d,e). In addition, cluster distributions in each sample were very similar between samples of the same conditions (Extended Data Fig. 1h).

Taken together, the characteristic population associated with response to muscle stretch appeared on pMI day 1 mainly within the IZ + BZ, decreased gradually in the IZ + BZ and sharply in the RZ on pMI day 7 and disappeared in both zones by pMI day 14.

### Spatial transcriptome uncovers spatiotemporal features

**Fig. 2:**
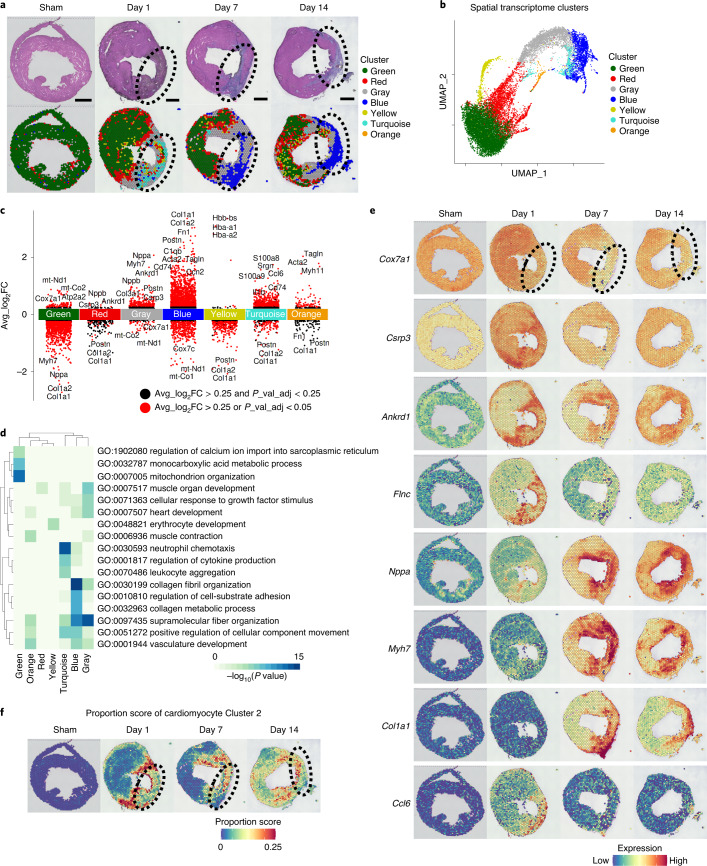
Spatial transcriptome analysis identifies spatiotemporally regulated cell clusters after MI. **a**, Representative images of H&E staining of heart cross-sections at each timepoint after MI surgery (above). Clusters found in Fig. 2b. were overlaid on the H&E images above (below). The areas surrounded by dotted lines indicate the IZ (sham, *n* = 1; pMI day 1, *n* = 3; pMI day 7, *n* = 3; and pMI day 14, *n* = 3). Scale bar, 1 mm. **b**, UMAP of all gene expression spots on the tissue from each timepoint. All spots (dots) were classified into seven clusters (named by the color of dots). **c**, Gene expression plot according to average log_2_ fold change (avg_log_2_FC) values in each cluster. Each dot represents a single gene. Names of genes characteristically upregulated and downregulated in each cluster were printed. Red indicates DEGs (avg_log_2_FC > 0.25 and adjusted *P* value (p_val_adj) < 0.05). Black indicates the other genes. **d**, Heat map showing the results of GO enrichment analysis for each cluster. Enrichment *P* values were generated by Metascape using cumulative hypergeometric distributions. **e**, Visualization of the expression level of representative genes in Clusters Green (*Cox7a1*), Red (*Csrp3*, *Ankrd1*), Gray (*Nppa*, *Myh7*), Blue (*Col1a1*) and Turquoise (*Ccl6*) at each timepoint. The areas surrounded by dotted lines indicate the IZ. **f**, Spatial heat maps showing the proportion score of cardiomyocyte Cluster 2. The areas surrounded by dotted lines indicate the IZ. Source data

**Extended Data Fig. 2:**
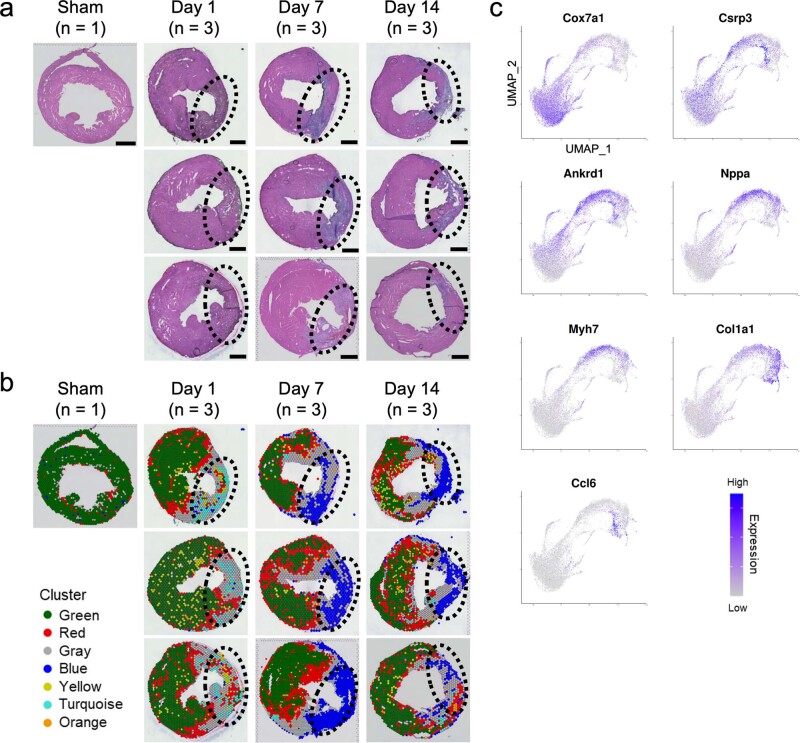
Clustering of spatial transcriptomics of the heart after MI. **a**, H & E staining at each time point (sham, n = 1; pMI day 1, n = 3; pMI day 7, n = 3; pMI day 14, n = 3). The area surrounded by dotted lines indicates the infarcted zone. **b**, Visualization of cell clusters at each time point. Each spot is colored with each cluster color. The area surrounded by dotted lines indicates the infarcted zone. **c**, Visualization of representative gene expression in clusters Green (*Cox7a1*), Red (*Csrp3*, *Ankrd1*), Gray (*Nppa*, *Myh7*), Blue (*Col1a1*), and Turquoise (*Ccl6*) on UMAP plot.

**Extended Data Fig. 3:**
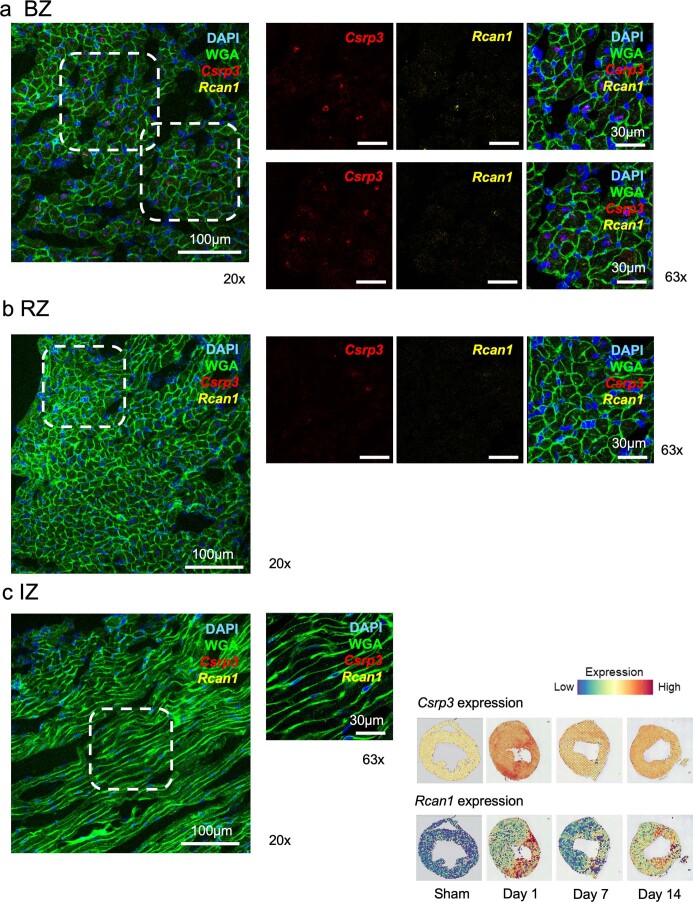
In situ hybridization of *Csrp3* and *Rcan1*. **a-c**, Representative images of *Csrp3* and *Rcan1* expression on pMI day 1 using in situ hybridization in BZ (a), RZ (b), IZ (c). Tissues in the area enclosed by the dashed line on 20× images are enlarged and shown on 63× images. Images show co-staining with DAPI (nucleus) and WGA (cell membrane). Scale bar, 100 μm (20×) and 30 μm (63×). Spatial gene expression of corresponding genes at each time point is shown in the bottom right corner.

**Extended Data Fig. 4:**
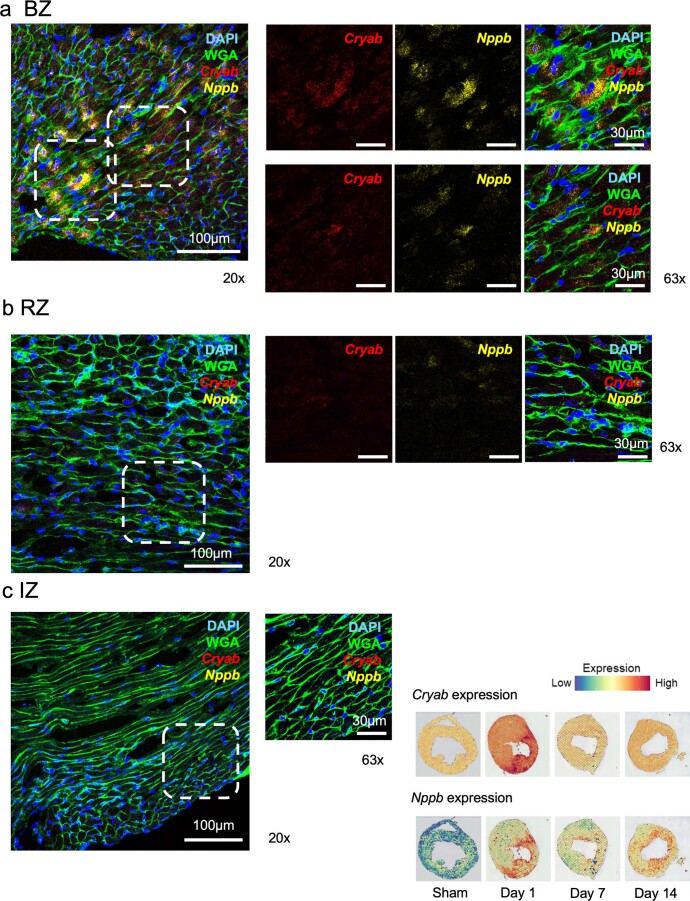
*In situ* hybridization of *Cryab* and *Nppb*. **a-c**, Representative images of *Cryab* and *Nppb* expression on pMI day 1 using in situ hybridization in BZ (a), RZ (b), IZ (c). Tissues in the area enclosed by the dashed line on 20× images are enlarged and shown on 63× images. Images show co-staining with DAPI (nucleus) and WGA (cell membrane). Scale bar, 100 μm (20×) and 30 μm (63×). Spatial gene expression of corresponding genes at each time point is shown in the bottom right corner. Source data

**Extended Data Fig. 5:**
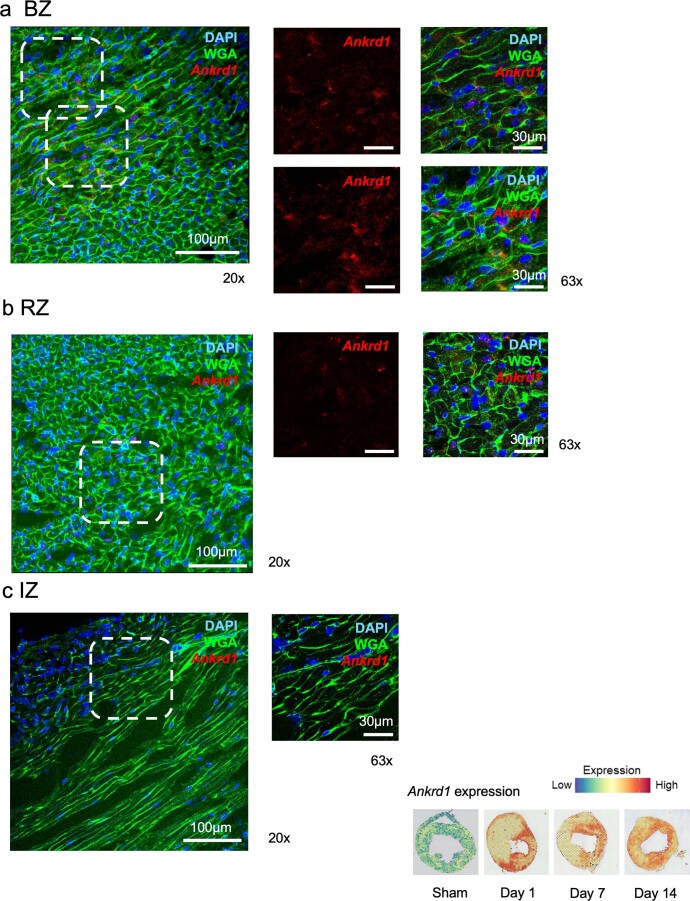
*In situ* hybridization of *Ankrd1*. **a-c**, Representative images of *Ankrd1* expression on pMI day 1 using in situ hybridization in BZ (a), RZ (b), IZ (c). Tissues in the area enclosed by the dashed line on 20× images are enlarged and shown on 63× images. Images show co-staining with DAPI (nucleus) and WGA (cell membrane). Scale bar, 100 μm (20×) and 30 μm (63×). Spatial gene expression of *Ankrd1* at each time point is shown in the bottom right corner.

**Extended Data Fig. 6:**
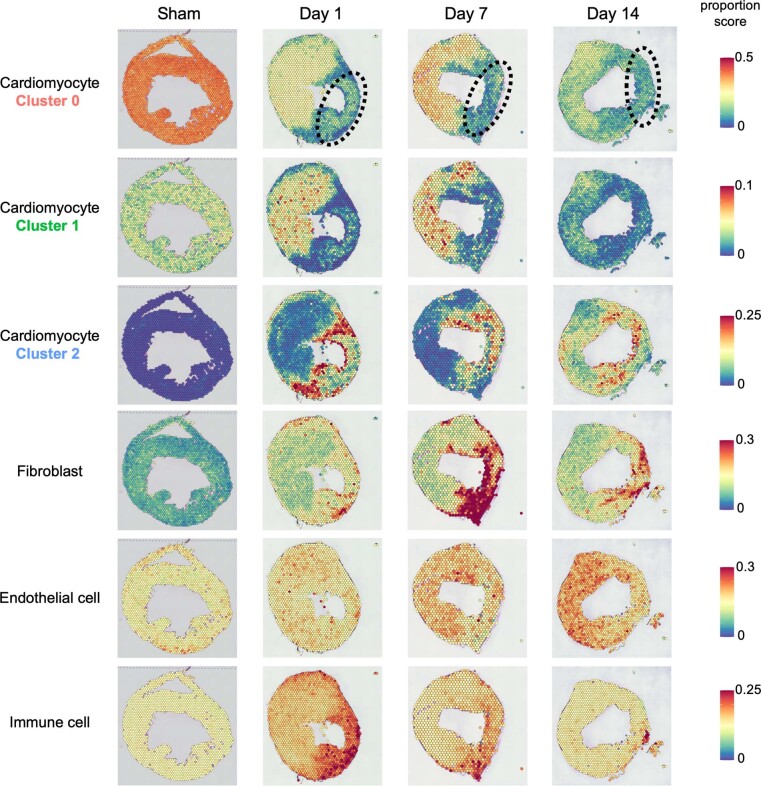
Cell type prediction analysis. Spatial heatmaps showing the proportion scores of each cell type.

To know the specific positional information of the cardiomyocyte clusters identified in our snRNA-seq analysis, we performed spatial transcriptome analysis. A mean of 1,679 spots (920–3,154 spots) from ten murine hearts (sham, *n* = 1; pMI day 1, *n* = 3; pMI day 7, *n* = 3; and pMI day 14, *n* = 3) were sequenced, and a median of 4,668 genes per spot (3,134–6,199) were detected. Figure 2a shows hematoxylin and eosin (H&E) staining of the hearts at each timepoint after MI surgery and illustrates the region clusters, which were revealed by clustering analysis of spatial transcriptomic data (Fig. 2b and Supplementary Tables 3 and 4). Although the IZ sizes were slightly different among mice, the characteristics of each clusterʼs distribution were similar and reproducible (Extended Data Fig. 2a,b). We also performed differentially expressed gene (DEG) analyses and GO enrichment analyses for each cluster (Fig. 2c,d). Spot regions of Cluster Green, characteristic for genes involved in the mitochondrial functional pathway (for example, *Atp2a2* and *Cox7a1*), occupied most of the sham hearts and the RZ of the infarcted hearts (Fig. 2a–e and Extended Data Fig. 2a–c). Cluster Blue, characterized by expressions of collagens and extracellular matrix genes (for example, *Co1a1* and *Postn*), was specifically localized within the IZ + BZ (Fig. 2a–e and Extended Data Fig. 2a–c). Spot regions of Cluster Red were mainly seen within the BZ of the infarcted hearts (Fig. 2a and Extended Data Fig. 2a,b). Among DEGs of Cluster Red, genes such as *Csrp3*, *Ankrd1* and *Nppb* (Fig. 2c), which were shared with those of Cluster 2 from the snRNA-seq analysis and were characterized by the function associated with the response to muscle stretch, were uniquely localized within the BZ of the infarcted hearts during the acute phase of MI (Fig. 2e). Spatially restricted expression patterns of these genes in cardiomyocytes were validated by RNA in situ hybridization (Extended Data Figs. 3–5). Cluster Gray appeared between Clusters Blue and Red and had characters common to both clusters, including a high expression of *Csrp3* (Fig. 2a–c,e and Extended Data Fig. 2b,c). Subsequently, we predicted the cell types composing each spot region by deconvolution analysis (Fig. 2f and Extended Data Fig. 6). The proportion score of the fibroblast cluster increased at the IZ from pMI day 7, whereas that of the immune cell cluster clearly increased at the IZ on pMI day 1. The proportion score of cardiomyocyte Cluster 2 was significantly high in many spots at the BZ on pMI day 1, although fewer spots showed high proportion score at the BZ on pMI day 7 and pMI day 14. This suggests that cardiomyocytes in Cluster 2 were mainly derived from the BZ in the acute phase of MI. To identify the role of the BZ in LV remodeling after MI, we focused on these BZ transcriptomes in the subsequent analysis.

### Weighted gene co-expression network analysis clarifies mechano-sensing gene expression in the BZ

**Fig. 3:**
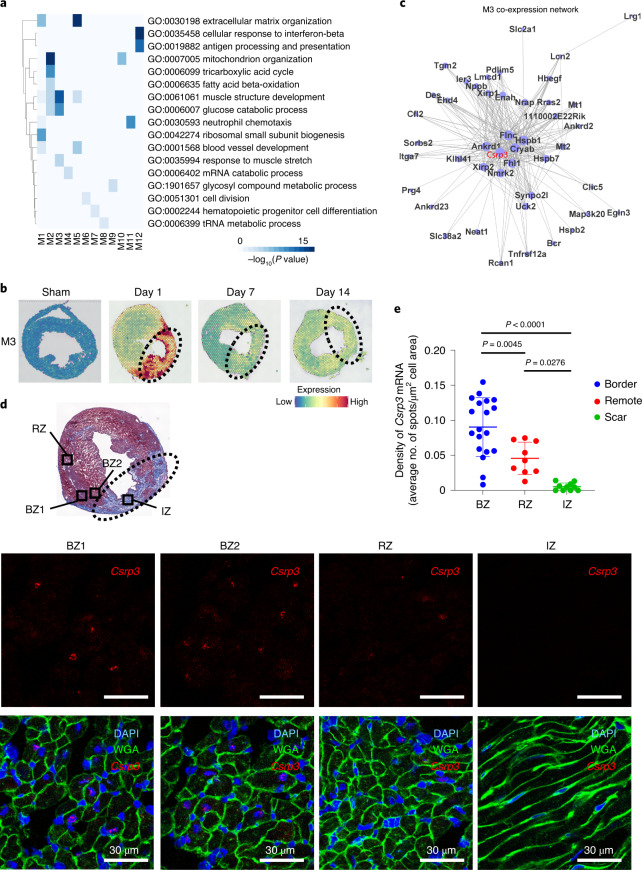
WGCNA of spatial transcriptomics reveals mechano-sensing genes as a distinct key factor expressed in the BZ after MI. **a**, Heat map showing the results of GO enrichment analysis for each module. Enrichment *P* values were generated by Metascape using cumulative hypergeometric distributions. **b**, Representative visualization of the module expression level of a characteristic gene module (M3) found by WGCNA at each timepoint. The areas surrounded by dotted lines indicate an IZ. **c**, Gene network of highly correlated genes in M3. Node size represents signed eigengene-based connectivity of a gene in a module. **d**, Masson’s trichrome staining of the heart cross-sections on day 1 after MI (above). Representative images of expression of *Csrp3* by in situ hybridization and merged images co-stained with DAPI (nucleus) and WGA (cell membrane) in two BZs (BZ1 and BZ2), RZ and IZ on pMI day 1 (below). The area surrounded by the dotted line indicates the IZ. Scale bar, 30 μm. **e**, Quantification of *Csrp3* mRNA molecules per cell. Nineteen images in the BZ, nine images in the RZ and ten images in the IZ were used to quantify the average number of *Csrp3* mRNA spots per cell. Data are shown as mean ± s.d. *P* values determined by a one-way ANOVA with Bonferroniʼs multiple comparison test are described in the figure. Source data

**Extended Data Fig. 7:**
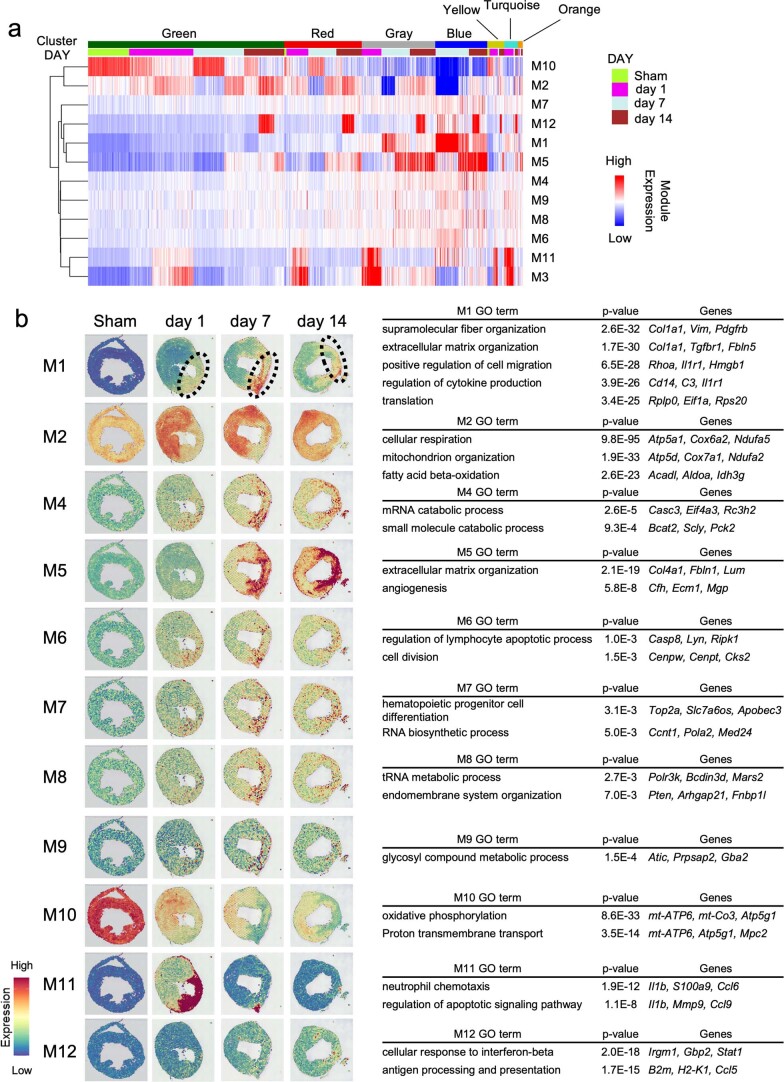
Weighted correlation network analysis of spatial transcriptomics of the hearts after MI. **a**, Expression heatmaps showing mean expression levels of each gene module (M1-M12) sorted by clusters found in Fig. 2b. Horizontal axis is colored by Clusters (1st row) and time points after MI (2nd row). **b**, Representative visualizations of module expression level (left) and tables showing the results of GO enrichment analysis (right) of each gene module (M1-12, except for M3) obtained following WGCNA at each time point (Sham, n = 1; pMI day 1, n = 3; pMI day 7, n = 3; pMI day 14, n = 3). Enrichment p values were generated by Metascape using cumulative hypergeometric distributions.

To clarify the spatiotemporally specific transcriptional network and find key factors in the BZ, we performed weighted gene co-expression network analysis (WGCNA) on the spatial transcriptomic data. WGCNA identified 12 gene modules, which were associated with clusters identified in Fig. 2 (Extended Data Fig. 7a and Supplementary Tables 5 and 6). Expressions of each gene module were regulated spatially and temporally (Extended Data Fig. 7b). Expressions of Module 1 (M1) and M5 genes, which encode collagen and extracellular matrix proteins (for example, *Col1a1*, *Vim* and *Fbln1*), were selectively upregulated in the IZ on pMI day 7 and pMI day 14 (Fig. 3a and Extended Data Fig. 7b). The deconvolution analysis suggests that these genes were mainly transcribed from fibroblasts (Extended Data Fig. 6). The M2 and M10 genes, which are associated with cellular respiration, fatty acid beta-oxidation and oxidative phosphorylation (for example, *Atp5a1*, *Cox7a1* and *Ndufa5*), were widely expressed in the sham hearts and the RZ of the infarcted hearts (Extended Data Fig. 7b) and were thought to be derived from cardiomyocytes in Cluster 0 or Cluster 1 in the snRNA-seq data (Extended Data Fig. 6). By contrast, the M3 genes were specifically expressed within the BZ of the pMI day 1 hearts (Fig. 3b). Most of the spots that highly expressed the M3 genes belonged to either Cluster Red or Cluster Gray on pMI day 1 (Extended Data Fig. 7a), and these genes were predicted to be transcribed mainly from Cluster 2 in the snRNA-seq data (Extended Data Fig. 6). The M3 co-expression network revealed mechano-sensing genes, such as *Csrp3*, *Ankrd1* and *Flnc*, as central genes (Fig. 3c), and response to muscle stretch is a GO term unique and specific for M3 (Fig. 3a). The dominant expression profiles of M3 genes in the BZ on pMI day 1 were validated using single-molecule RNA in situ hybridization (Fig. 3d,e and Extended Data Figs. 3–5). Because *Csrp3* is a well-known mechano-sensing gene and is located at the center of the BZ transcriptome (M3), we focused on the function of *Csrp3* at the BZ and its role in LV remodeling after MI.

### *Csrp3* expression in BZ cardiomyocytes prevents LV remodeling

**Fig. 4:**
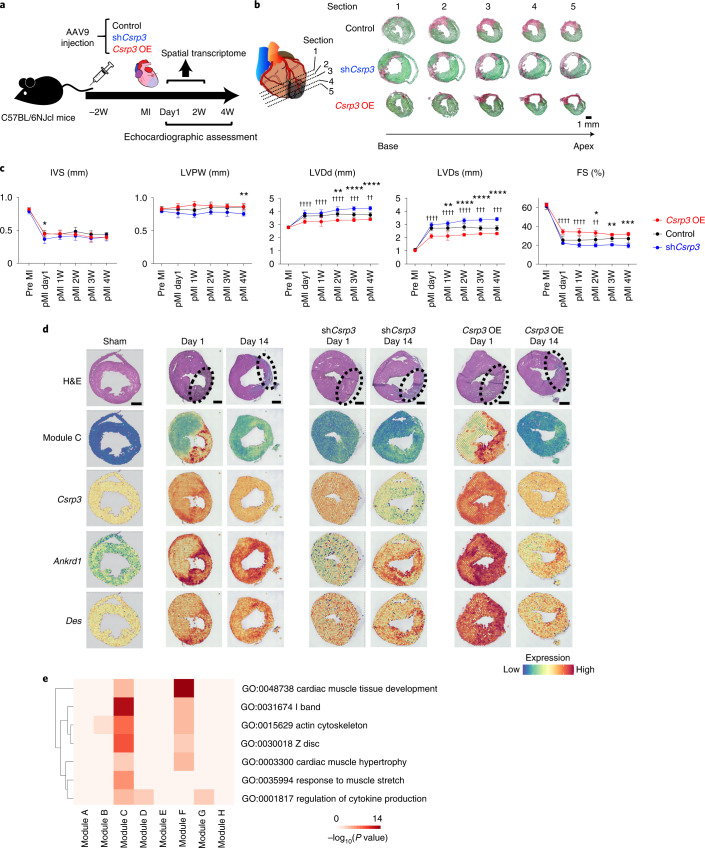
*Csrp3* expressed in cardiomyocytes in the BZ adaptively limits adverse LV remodeling after MI. **a**, Experimental design for testing the effect of AAV9-sh*Csrp3* and AAV9-*Csrp3* vectors. **b**, Representative histological sections through multiple levels of the heart (base toward the apex) with Picrosirius Red/Fast Green dye staining showing the scar tissue in pink and the healthy myocardium in green. Control, AAV9-sh*Csrp3*-injected and AAV9-*Csrp3-*injected hearts on pMI day 14 are shown. Scale bar, 1 mm. **c**, Echocardiographic assessment of the heart of control and AAV9-sh*Csrp3-*injected mice after MI surgery. Data are shown as mean ± s.d. Control, *n* = 12; sh*Csrp3*, *n* = 5; and *Csrp3* OE, *n* = 5. *P < 0.05 (control versus sh*Csrp3*), **P < 0.01 (control versus sh*Csrp3*), ***P < 0.005 (control versus sh*Csrp3*), ****P < 0.001 (control versus sh*Csrp3*), ^††^*P* < 0.01 (control versus *Csrp3* OE), ^†††^*P* < 0.005 (control versus *Csrp3* OE) and ^††††^*P* < 0.001 (control versus *Csrp3* OE); significance was determined using a two-way ANOVA with Bonferroniʼs multiple comparison test. **d**, Representative images of H&E staining of whole heart, module expression level of Module C and gene expressions of *Csrp3*, *Ankrd1* and *Des* at each timepoint after MI surgery with or without AAV9-sh*Csrp3* or AAV9-*Csrp3*. The areas surrounded by dotted lines indicate the IZ (sham, *n* = 1; pMI day 1, *n* = 3; pMI day 14, *n* = 3; AAV9-sh*Csrp3* on pMI day 1, *n* = 1; AAV9-sh*Csrp3* on pMI day 14, *n* = 1; AAV9-*Csrp3* on pMI day 1, *n* = 1; and AAV9-*Csrp3* on pMI day 14, *n* = 1). Scale bar, 1 mm. **e**, Heat map showing the results of GO analysis with biological process and cellular component enriched in Module C. Enrichment *P* values were generated by Metascape using cumulative hypergeometric distributions. FS, fractional shortening; IVS, interventricular septum; OE, overexpression; LVDs, left ventricular end-systolic dimension; LVDd, left ventricular end-diastolic dimension; LVPW, left ventricular posterior wall. W, weeks. Source data

**Extended Data Fig. 8:**
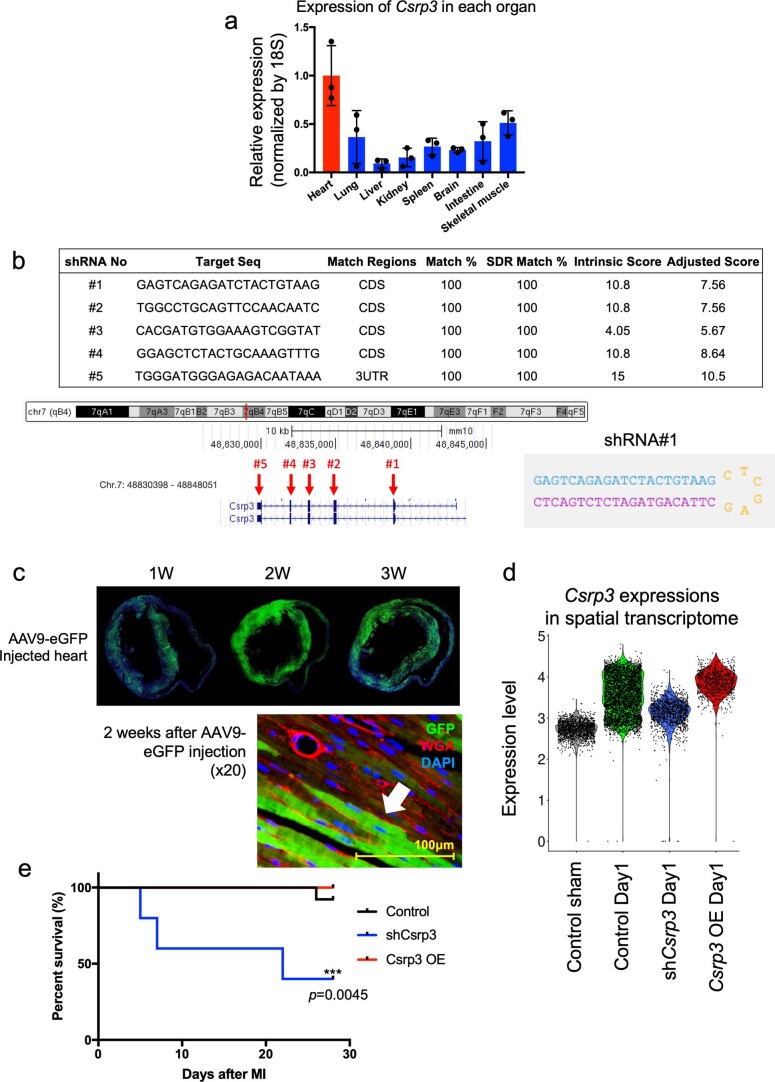
Analysis of *Csrp3* knockdown and overexpression mice. **a**, mRNA expression levels of *Csrp3* in various mouse organs were assessed using real-time qPCR (n = 3 at each organ). Data are shown as mean ± SD. **b**, Design of sh*Csrp3* knockdown vector. Each shRNA was designed using ‘The RNAi consortium database’ (by Broad Institute) and #1 was chosen in this study. **c**, Images of Egfp expression within heart sections collected 1 week, 2 weeks, and 3 weeks after injection of AAV9-*Egfp*. This experiments were independently repeated two times to confirm that the similar expression level of Egfp were observed. **d**, Relative *Csrp3* expression levels in the hearts of each group (control, AAV9-sh*Csrp3*, and AAV9-*Csrp3*). **e**, Survival curves comparing Control (n = 13), AAV9-sh*Csrp3* injected mice (n = 5), and AAV9-*Csrp3* injected mice (n = 5) after MI surgery. P = 0.0045; statistical analysis was performed using the Gehan–Breslow–Wilcoxon test.

Among many organs, the *Csrp3* expression is predominant in the heart, specifically in cardiomyocytes (Extended Data Fig. 1b and Extended Data Fig. 8a). To clarify the roles of *Csrp3* and its upregulation within the BZ of the pMI day 1 heart, we overexpressed *Csrp3* and short hairpin RNA (shRNA) against *Csrp3* using AAV9 vectors (Extended Data Fig. 8b). A previous report^19^ as well as our preliminary experiment (Extended Data Fig. 8c) showed that a gene delivered by AAV9 vector was highly expressed 2 weeks after viral injection. Because *Csrp3* is highly expressed in the acute phase of MI, we injected the vectors into mice through their tail veins 2 weeks before MI surgery and performed transthoracic echocardiography to analyze their cardiac function (Fig. 4a). Transduction of the shRNA significantly reduced expression of *Csrp3* in the heart, whereas overexpression of *Csrp3* significantly increased its expression (Extended Data Fig. 8d). The local elevation of *Csrp3* expression within the BZ was also repressed on pMI day 1 (Fig. 4d) by the injection of sh*Csrp3*-AAV9. Compared to controls, the AAV9-sh*Csrp3-*injected mice showed enlarged left ventricles and reduced LV contraction after MI, whereas *Csrp3* overexpression tended to alleviate the disease phenotype (Fig. 4b,c). Although there was no statistical significance in survival rates between controls and *Csrp3-*overexpressed mice, the AAV9-sh*Csrp3-*injected mice showed poorer outcomes than the control mice (Extended Data Fig. 8e).

**Extended Data Fig. 9:**
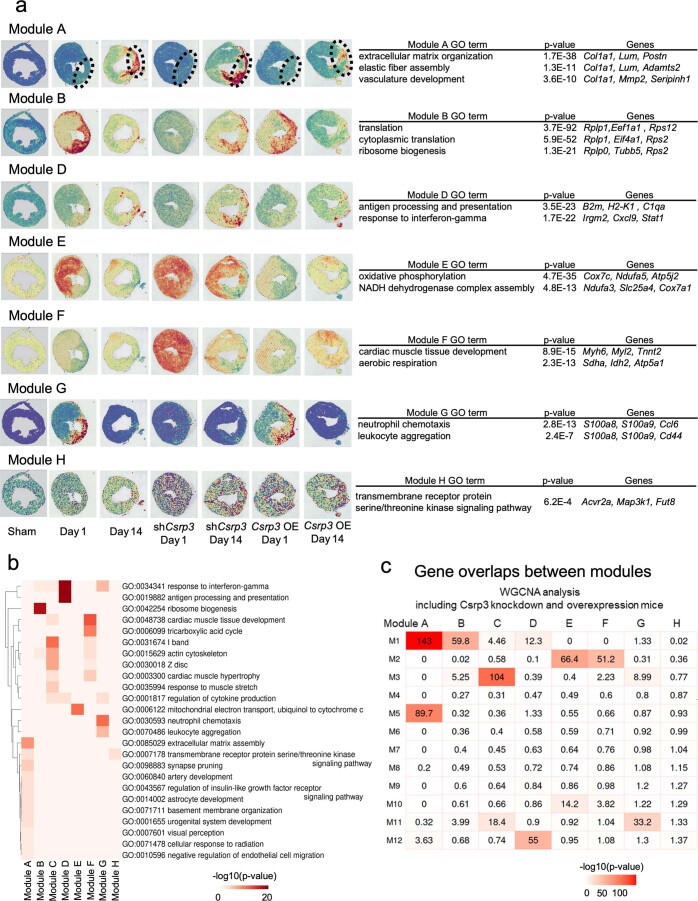
Spatial transcriptome analysis including Csrp3 knockdown and overexpression mice. **a**, Representative visualizations of module expression levels and tables showing the results of GO biological process enrichment analysis of each gene module (Module A-H, except for Module C) obtained following WGCNA including *Csrp3* knockdown and overexpressing mice at each time point (sham, n = 1; pMI day 1, n = 3; pMI day 14, n = 3; pMI day 1 with AAV9-sh*Csrp3*, n = 1; pMI day 14 with AAV9-sh*Csrp3*, n = 1; pMI day 1 with AAV9-*Csrp3*, n = 1; and pMI day 14 with AAV9-*Csrp3*, n = 1). Enrichment p values were generated by Metascape using cumulative hypergeometric distributions. **b**, Heatmap showing the results of gene ontology enrichment analysis for each cluster. Enrichment p values were generated by Metascape using cumulative hypergeometric distributions. **c**, Heatmap showing the significance of gene overlaps between modules following WGCNA analysis of sham, n = 1; pMI day 1, n = 3; pMI day 7, n = 3; and pMI day 14, n = 3, and modules by WGCNA of sham, n = 1; pMI day 1, n = 3; pMI day 14, n = 3; pMI day 1 with AAV9-sh*Csrp*3, n = 1; pMI day 14 with AAV9-sh*Csrp3*, n = 1; pMI day 1 with AAV9-*Csrp3*, n = 1; and pMI day 14 with AAV9-*Csrp3*, n = 1. The table is colored by -log10(P-value), obtained using the Fisher’s exact test.

**Extended Data Fig. 10:**
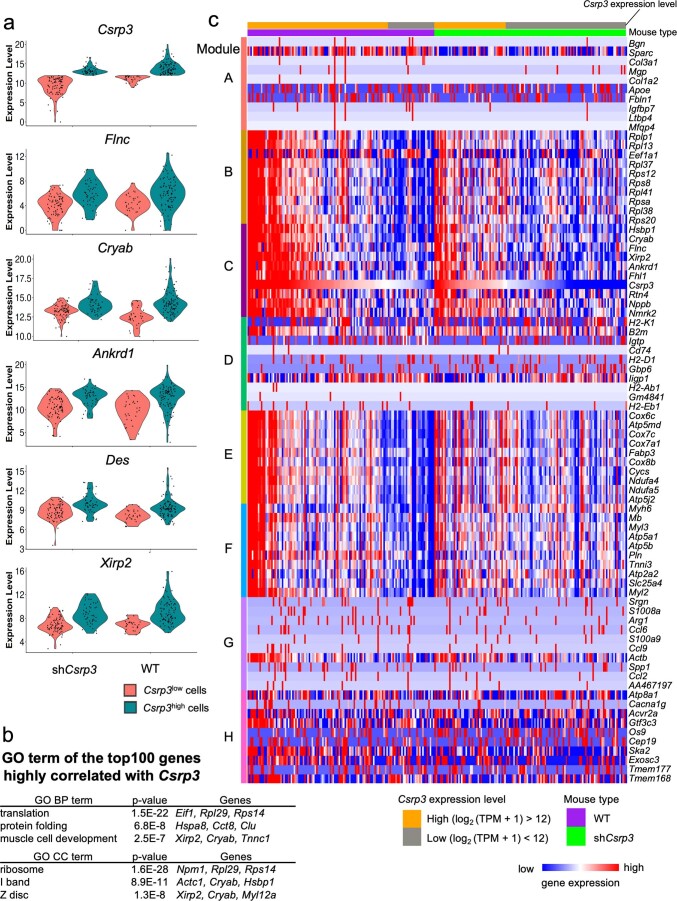
single-cardiomyocyte RNA-seq of wild-type (WT) and *Csrp3* knockdown mice. **a**, VlnPlot showing *Csrp3* gene expressions, and the representative genes associated with Z-disc from single-cardiomyocyte RNA-seq on pMI day 1 of WT and *Csrp3* knockdown (sh*Csrp3*) mice (WT, n = 2; sh*Csrp3*, n = 2). Plots are divided into WT/sh*Csrp3* and *Csrp3*-high/low cardiomyocytes. Expression levels were calculated by log_2_ (TPM + 1). **b**, Tables showing the results of GO enrichment analysis of the top 100 genes whose expression is highly correlated with *Csrp3* expression. GO, gene ontology; BP, biological process; CC, cellular component. Enrichment p values were generated by Metascape using cumulative hypergeometric distributions. **c**, Heatmap showing the expression levels of the representative genes from each module. Cells are sorted by *Csrp3* expression levels and grouped by mouse types.

We also performed spatial transcriptomic analyses of both the *Csrp3* knockdown and the *Csrp3-*overexpressed hearts and performed another WGCNA (Extended Data Fig. 9a,b and Supplementary Table 7). Among the eight modules obtained (Modules A–H), Module C corresponded to M3 (Extended Data Fig. 9c), which is the distinct module seen in the BZ on pMI day 1 (Fig. 3a–c). In the BZ of the pMI day 1 hearts, expression of Module C genes, including *Ankrd1* and *Des*, was upregulated both in the control and *Csrp3-*overexpressed hearts, whereas expression of Module C was not upregulated in the *Csrp3* knockdown heart (Fig. 4d). Module C was characterized by genes involved in response to muscle stretch and was composed of various genes expressed around the Z-disc (Fig. 4d,e). Furthermore, we performed single-cardiomyocyte RNA-seq of the IZ + BZ cardiomyocytes from the hearts of wild-type (WT) mice and *Csrp3* knockdown mice on pMI day 1 to evaluate the expression changes of genes associated with Z-disc after MI (WT, *n* = 2; *Csrp3* knockdown, *n* = 2). We separated cardiomyocytes into two groups according to the *Csrp3* expression levels: *Csrp3*-high cardiomyocytes and *Csrp3*-low cardiomyocytes. Z-disc-associated genes, such as *Ankrd1*, *Flnc* and *Des*, were downregulated in the *Csrp3*-low cardiomyocytes (Extended Data Fig. 10a). GO enrichment analysis showed that genes whose expression was highly correlated with the *Csrp3* expression were related to muscle structure development and Z-disc (Extended Data Fig. 10b). In addition, Module C genes, which were observed within the BZ on pMI day 1, were highly expressed in the *Csrp3*-high cardiomyocytes (Extended Data Fig. 10c). Thus, *Csrp3* expression is associated with the expression of other mechano-sensing-associated and Z-disc-associated genes.

## Discussion

A spatial transcriptome technology is a powerful tool to deepen the understandings of spatial pathophysiology in various tissues. Because MI is caused by occlusion of a coronary artery, the distance from the occlusion site significantly affects the response of each region. Therefore, a spatial perspective is important to clarify the pathophysiological processes after MI. In the present study, we comprehensively investigated the spatiotemporal gene expression profiles of the heart after MI and identified several gene programs that were precisely regulated in a spatiotemporal manner. We revealed that transcriptional responses of mechano-sensing genes at the BZ are crucial to prevent LV remodeling after MI.

Previous studies examined the expression of RNA or proteins using myocardial tissues around the BZ dissected from infarcted hearts and reported that expression of genes associated with angiogenesis, inflammation, apoptotic response and B-type natriuretic peptide was highly upregulated within the BZ^10,20–23^. These analyses were performed using bulk RNA-seq or scRNA-seq with the ambiguous spatial localization of the BZ without precise spatial information. Some studies have recently tried to clarify the spatial pathophysiology using the healthy and diseased hearts. In a previous study, we spatially quantified gene expression of the murine heart after pressure overload at the single-cell level using in situ RNA hybridization and identified spatially heterogenous *Myh7* expression among cardiomyocytes under pressure overload^24^. Mohenska et al.^25^ provided comprehensive spatial transcriptomics data of healthy adult murine hearts using micro-dissected 18 sections of heart regions. Their study unveiled specific genes in each section, but the resolution was much lower than single-cell level. Lacraz et al.^14^ investigated the spatial transcriptional changes around the BZ in an infarcted heart using Tomo-seq and identified SOX9 as an important transcriptional regulator that activates fibrosis-related gene expression in *Col1*-positive cardiac fibroblasts under ischemia. They used 48 cryo-sections with a thickness of 80 µm and a width of 2.5 mm. The thickness was thin enough to observe gradual transcriptional changes around the BZ; however, the width was not narrow enough to analyze the function of each cell type in each region, and 48 slices were also not enough to evaluate the entire area, including the IZ and the RZ. Asp et al.^26^ combined scRNA-seq, spatial transcriptomics and in situ sequencing to generate a three-dimensional gene expression atlas of the developing human heart. Their study profiled spatiotemporal gene expression patterns and analyzed roles of diverse cell types in human cardiogenesis. Kuppe et al. performed integrative analysis of scRNA-seq, scATAC-seq and spatial transcriptomics using both healthy and infarcted human heart tissues (preprint at https://www.biorxiv.org/content/10.1101/2020.12.08.411686v1.full).

As mentioned above, some studies regarding spatial transcriptome of heart development or heart diseases have been reported. However, none of them has clarified the precise molecular mechanisms of heart disease because of some limitations, including low resolution and narrow analysis range. We performed a spatiotemporal transcriptome analysis with high resolution in entire cross-sections of time-dependent diseased hearts. In this study, we combined spatial transcriptomics and snRNA-seq to obtain spatiotemporal expression profiles after MI at the single-cell level and identified mechano-sensing genes, including *Csrp3*, to be expressed in the BZ at an early phase after MI as an adaptive regulator of LV remodeling. Because there is no study to serially perform scRNA-seq/snRNA-seq and spatial transcriptome analysis over time after MI, our integrative datasets provide a publicly available resource to further deepen understanding of the pathogenesis of MI. Besides, compared to spatial transcriptome analysis using the sections from limited areas of the human heart^14^, our study provides the spatial transcriptome landscape of the whole murine heart. Although we focused, in the present study, on the transcriptional signature in the BZ during the acute phase after MI, our dataset would be a valuable resource that could be applied to a variety of future studies for elucidating the pathogenesis of MI.

Our spatial transcriptomic analysis clearly revealed that expression of mechano-sensing genes, including *Csrp3*, was upregulated in the acute phase after MI in the BZ. Expression of *Csrp3* was reported to be upregulated in cardiomyocytes by mechanical stretch^27^ as well as in the heart by pressure overload^28,29^. A recent study using echocardiography for a porcine MI model showed that the end-diastolic mean wall stress of the BZ is increased from acute phase of MI and that myocardial stiffness of this region also reaches the plateau by 14 days after MI^30^, suggesting that cardiomyocytes activate the expression of *Csrp3* in response to mechanical stresses caused by MI. CSRP3 is known to be located in the Z-disc and acts as a stretch sensor by interacting with various proteins, such as titin-cap (Tcap)^31^, Ankrd1^32^, PKCα^32^, calcineurin^33^ and α-actinin^34,35^. Our results demonstrated that the downregulation of *Csrp3* aggravates cardiac dysfunction after MI, whereas the upregulation of *Csrp3* ameliorates it. In addition, *Csrp3* knockdown induced downregulation of other mechano-sensing genes especially localized near the Z-disc within the BZ of the acute phase after MI (Fig. 4d,e and Extended Data Fig. 10). The mutations of various genes encoding the proteins around the Z-disc, such as *ANKRD1*, *DES*, *FLNC* and *CSRP3*, are known to cause abnormal responses to mechanical stress, resulting in heart failure. These results and observations suggest the possibility that upregulation of *Csrp3* expression in cardiomyocytes at the BZ prevents cardiac remodeling after MI and is involved in upregulation of other mechano-sensing genes. Considering that *CSRP3* is significantly downregulated in chronic human heart failure^36,37^, *CSRP3* could be a therapeutic target for chronic heart failure as well as for MI.

In summary, we performed an integrative analysis of snRNA-seq and spatial transcriptome using MI hearts obtained in a time-serial manner. We revealed that gene expression was precisely regulated in a spatiotemporal manner after MI and that mechano-sensing genes upregulated in the BZ during the acute phase after MI play a preventive role against post-MI LV remodeling.

## Methods

### Animal models

All animal experiments were approved by the University of Tokyo Ethics Committee for Animal Experiments and strictly adhered to the guidelines for animal experiments of the University of Tokyo (approval no. P14-106). All WT C57BL/6 male mice were purchased from CLEA Japan. Mice were housed in cages with a maximum of six mice per cage in a specific-pathogen-free, temperature-controlled vivarium under a 12-hour light/dark cycle with ad libitum access to food and water. Ambient room temperature was regulated at 73 ± 5 °F, and humidity was controlled at 50 ± 10%.

### Operation of MI model and echocardiography

MI was induced as previously described^38^. Nine- to 11-week-old male mice were used for all experiments. Sample size was determined based on the experimental results of our previous study^39^. Mice were anesthetized by inhalation of 2% isoflurane. MI was induced by ligation of the left anterior descending artery. Mice that failed to develop MI or died within 1 day after the operation were excluded from the analysis. Transthoracic echocardiography was performed on conscious mice, using a Vevo 2100 imaging system (FUJIFILM VisualSonics). To minimize variation in the data, the cardiac function was assessed only when the heart rate was 600–700 beats per minute. M-mode echocardiographic images were obtained from a longitudinal view to measure the size and function of the left ventricle. Significance was determined by a two-way ANOVA with Bonferroniʼs multiple comparison test using GraphPad Prism 7.0e.

### snRNA-seq of cardiomyocytes using murine heart

For snRNA-seq of cardiomyocytes, cardiomyocytes were isolated using Langendorff perfusion from the left ventricle as previously described^29^. Langendorff perfusion enzymatic dissociation was performed with 35 ml of enzyme solution (type 2 collagenase 1 mg/ml (Worthington Biochemical), protease type XIV 0.05 mg/ml (Sigma-Aldrich), NaCl 130 mM, KCl 5.4 mM, MgCl_2_ 0.5 mM, NaH_2_PO_4_ 0.33 mM, D-glucose 22 mM and HEPES 25 mM pH 7.4) pre-heated to 37 °C, at a rate of 3 ml min^−1^. To neutralize enzymatic activity, FBS was added to the solution to a final concentration of 0.2% (v/v). After perfusion with digestion buffer, the left ventricle was dissected according to sectional regions (IZ + BZ versus RZ), and cardiomyocytes were subsequently isolated. Cell suspensions were filtered through a 100-μm nylon mesh cell strainer and centrifuged at 100*g* for 2 minutes, after which the supernatant was discarded. To prevent hypercontraction, the cardiomyocytes were resuspended in medium (NaCl 130 mM, KCl 5.4 mM, MgCl_2_ 0.5 mM, NaH_2_PO_4_ 0.33 mM, D-glucose 22 mM, HEPES 25 mM, FBS 0.2% pH 7.4) containing a low concentration of calcium (0.1 mM). Nuclei were isolated immediately after the collection of cardiomyocytes using Minute Detergent-Free Single Nuclei Isolation Kit (NI-024, Invent Biotechnologies) according to the manufacturer’s instructions. Single-nucleus cDNA libraries were generated using 5,000 isolated nuclei and the Chromium 3′ v3 chemistry kit (10x Genomics) according to the manufacturer’s instructions.

### snRNA-seq data analysis of MI model mice

Raw FASTQ files were processed by sample with the Cell Ranger software (version 6.1.1, 10x Genomics), against the mm10 reference genome ‘refdata-gex-mm10-2020-A’ with ‘include-introns’ and ‘--force-cells=5000’. Raw counts were used as input for the Seurat R package (version 4.1.1). We removed the low-quality nuclei with fewer than 500 detected genes or with a high percentage of mitochondrial genes (higher than 60%). After quality control, a total of 12,813 nuclei were used for the downstream analysis. Next, mitochondrial RNA genes were filtered out as well to exclude transcripts originating from outside the nucleus and to avoid biases introduced by the isolation of nuclei. We performed log-normalization using the NormalizeData function, 10,000, and identified highly variable features of each dataset with the FindVariableFeatures (nfeatures = 2000) function. Then, we integrated all datasets by using FindIntegrationAnchors and IntegratedData. After that, we performed a linear regression on all genes using ScaleData. We used RunPCA for dimensional reduction and used FindClusters for graph-based clustering. We performed uniform manifold approximation and projection (UMAP) with RunUMAP. We detected nuclei population with highly *Tnnt2* expression as a cardiomyocyte subset (Cluster 0). We performed ScaleData, RunPCA, FindClusters and RunUMAP against the cardiomyocyte subset. DEGs were detected by using FindMarkers (log_2_fc.threshold > 0.25 and p_val_adj < 0.05 (adjusted *P* value based on Bonferroniʼs correction)). Top DEGs were presented according to the log_2_ fold change of the average expression (avg.log_2_FC). The top 150 marker genes of each cluster or all marker genes if fewer than 150 were used for GO enrichment analysis. GO enrichment analysis was performed using Metascape with GO biological processes^40^.

### Spatial transcriptome analysis of mice

Frozen samples were embedded in OCT (TissueTek) and cryo-sectioned at −14 °C (Leica, CM1860). Sections were placed on chilled Visium Spatial Gene Expression Slides (2000233, 10x Genomics) and adhered by warming the back of the slide. Tissue sections were then fixed in chilled methanol and stained according to the Visium Spatial Gene Expression user guide (CG000239 Rev D, 10x Genomics). For gene expression samples, tissue was permeabilized for 15 minutes, which was selected as the optimal time based on tissue optimization time course experiments (CG000238 Rev D, 10x Genomics). Bright-field histology images of H&E staining were taken using a ×10 objective on a BZ-X700 microscope (Keyence). Raw images were stitched together using BZ-X analyzer software (Keyence) and exported as TIFF files.

Libraries were prepared according to the Visium Spatial Gene Expression user guide (CG000239 Rev D, 10x Genomics) and sequenced on a NovaSeq 6000 System (Illumina) using a NovaSeq S4 Reagent Kit (200 cycles, 20027466, Illumina), at a sequencing depth of approximately 250–400 million read pairs per sample. Sequencing was performed using the following read protocol: read 1, 28 cycles; i7 index read, ten cycles; i5 index read, ten cycles; and read 2, 91 cycles.

### Spatial transcriptome data processing

Raw FASTQ files and histology images were processed by sample using the Space Ranger software (version 1.2.1, 10x Genomics), against the Cell Ranger mm10 reference genome ‘refdata-gex-mm10-2020-A’. Raw counts were used as input for the Seurat R package (version 4.1.1), and log-normalization was implemented separately for each dataset and integrated by using the FindIntegrationAnchors and IntegratedData functions. Then, we performed a linear regression on all genes using ScaleData. We used RunPCA for dimensional reduction and FindClusters for graph-based clustering. We performed UMAP with RunUMAP. DEGs were detected by using FindMarkers in Seurat (log2fc.threshold > 0.25 and p_val_adj < 0.05 (adjusted *P* value based on Bonferroniʼs correction)). Top DEGs were presented according to the log_2_ fold change of the average expression (avg.log_2_FC). The top 100 marker genes of each cluster or all marker genes if fewer than 100 were used for GO enrichment analysis. GO enrichment analysis was performed using Multiple Gene Lists of Metascape with GO biological processes^40^. In the analysis of cell type proportion in each spot, we used the CARD (version 1.0) R package^41^. As reference data, from snRNA-seq analysis, we used three clusters of cardiomyocyte subcluster; cluster 1 expressing *Ptprc* as immune cell; clusters 2 and 8 expressing *Pecam1* as endothelial cell; clusters 3, 4 and 5 expressing *Col1a1* as fibroblast; cluster 6 expressing *Rgs5* as pericyte; and cluster 9 expressing *Upk3b* as pericardial cell (Extended Data Fig. 1a,b). We deconvoluted the spatial transcriptome data and calculated the proportion scores with CARD_deconvolution.

### WGCNA of WT mice

We performed analysis on sham (*n* = 1), pMI day 1 (*n* = 3), pMI day 7 (*n* = 3) and pMI day 14 (*n* = 3) WT mice. Counts per million (CPMs) were used as input for the WGCNA analysis (version 1.69)^42^. All genes expressed at more than 1,600 spots (approximately 10% of all spots) were used for analysis. The soft power threshold was analyzed with the pickSoftThreshold function and was applied to construct a signed network and calculate module eigengene expression using the blockwiseModules function. Modules with fewer than 15 genes were merged to their closest larger neighboring module. To visualize the weighted co-expression networks, Cytoscape (version 3.8.0)^43^ with the ‘prefuse force-directed layout’ was used. Signed eigengene-based connectivity of a gene in a module reflected the node size. GO enrichment analysis of genes in each module was performed using Metascape with GO biological processes^40^. To compare among modules, the top 100 genes or all genes if fewer than 100 were applied into Multiple Gene Lists of Metascape with GO biological process.

### WGCNA of WT, knockdown and overexpressing mice

We performed analysis on sham (*n* = 1), pMI day 1 (*n* = 3) and pMI day 14 (*n* = 3) WT mice; *Csrp3* knockdown mice at pMI day 1 (*n* = 1) and pMI day 14 (*n* = 1); and *Csrp3-*overexpressing mice of pMI day 1 (*n* = 1) and pMI day 14 (*n* = 1). CPMs were used as input for the WGCNA analysis (version 1.69)^42^. All genes expressed at more than 1,600 spots (approximately 10% of all spots) were used for analysis. Statistically significant differences in allocated genes between groups were assessed with Fisher’s exact test. GO enrichment analysis of genes in each module was performed using Metascape with GO biological processes^40^. To compare among modules, the top 100 genes or all genes if fewer than 100 were applied into Multiple Gene Lists of Metascape with GO biological process and cellular components.

### Single-cardiomyocyte RNA-seq analysis of WT and *Csrp3* knockdown mice

We performed single-cardiomyocyte RNA-seq from the hearts of WT and *Csrp3* knockdown mice on pMI day 1 (WT, *n* = 2; *Csrp3* knockdown, *n* = 2). Cardiomyocytes were isolated using Langendorff perfusion from the left ventricle as previously described^29^. After perfusion of digestion buffer, the left ventricle was dissected according to sectional regions (IZ + BZ, and RZ), and cardiomyocytes were subsequently isolated. IZ + BZ includes the infarct zone and 2mm of its lateral margin, and RZ is the other area. Cell suspensions were filtered through a 100-μm nylon mesh cell strainer and centrifuged at 100*g* for 2 minutes, after which the supernatant was discarded. To prevent hypercontraction, the cardiomyocytes were resuspended in medium (NaCl 130 mM, KCl 5.4 mM, MgCl_2_ 0.5 mM, NaH_2_PO_4_ 0.33 mM, D-glucose 22 mM, HEPES 25 mM, FBS 0.2% pH 7.4) containing a low concentration of calcium (0.1 mM). Rod-shaped live cardiomyocytes (viability of cardiomyocytes was ≥80% for all timepoints) were collected immediately with a 0.2–2-µl pipette (sample volume, 0.5 µl) and incubated in lysis buffer.

Single-cardiomyocyte cDNA libraries were generated using the Smart-seq2 protocol^44^, and the efficiency of reverse transcription was assessed by examining the cycle threshold (Ct) values of control genes (*Tnnt2*) from quantitative real-time polymerase chain reaction (qRT–PCR) using a CFX96 Real-Time PCR Detection System (Bio-Rad), and the distribution of cDNA fragment lengths was assessed using LabChip GX (PerkinElmer) and/or TapeStation 2200 (Agilent Technologies). The following primer sets were used for qRT–PCR: *Tnnt2* mRNA forward: TCCTGGCAGA GAGGAGGAAG; *Tnnt2* mRNA reverse: TGCAGGTCGA ACTTCTCAGC. A Ct value of 25 was set as the threshold. The sequencing libraries were subjected to paired-end 150-bp RNA-seq on a NovaSeq 6000 (Illumina).

### Single-cardiomyocyte RNA-seq data processing

Raw sequencing reads from single-cardiomyocyte RNA-seq libraries were trimmed to remove adapter sequences and low-quality bases using fastp-0.21.0 (ref. ^45^) with the parameters ‘--cut_tail --cut_tail_window_size 10 --cut_tail_mean_quality 30 --length_required 100’. The reference transcript data and the gene annotation file for the mouse were downloaded from GENCODE (release 26, https://www.gencodegenes.org/)^46^. The clean reads were aligned to the mouse genome (mm10) using STAR (version 2.7.8a)^47^. The reads aligned to the exon were counted using featureCounts^48^. Transcripts per million (TPM) normalization was calculated with reads mapped to the nuclear genome. We removed the low-quality cardiomyocytes with fewer than 4,000 detected genes, which were used for the downstream analysis (WT, 125 cardiomyocytes; sh*Csrp3*, 129 cardiomyocytes). We separated cardiomyocytes into two groups according to the *Csrp3* expression levels: *Csrp3*-high cardiomyocytes (log_2_(TPM + 1) > 12) and *Csrp3*-low cardiomyocytes (log_2_(TPM + 1) < 12). Gene expression correlation with *Csrp3* expression was calculated using Pearson correlation. The top 100 highly correlated genes sorted by Pearson correlation coefficient were analyzed for GO enrichment analysis using Metascape with GO biological processes and cellular components.

### AAV9-sh*Csrp3* and AAV9-*Csrp3* infection

The AAV vectors were prepared by VectorBuilder (https://en.vectorbuilder.com) according to established procedures^49^. In brief, AAV vectors of serotypes 2 and 9 were generated in HEK293T cells, using triple-plasmid co-transfection for packaging. Viral stocks were obtained by CsCl_2_ gradient centrifugation. Titration of AAV viral particles was performed using real-time PCR quantification of the number of viral genomes, measured as cytomegalovirus (CMV) copy number. The viral preparations had a titer between 1 × 10^12^ and 5 × 10^12^ genome copies (GC) per milliliter. Viruses were administered in 100-μl saline via tail vein injections. A 3 × 10^11^ GC dose of AAV9-e*GFP* or 3 × 10^11^ GC doses of AAV9-sh*Csrp3* or AAV9-*Csrp3* were administered to the uninjured mice 2 weeks before MI surgery.

### RNA in situ hybridization

For RNA fluorescence in situ hybridization, the RNAscope system (Advanced Cell Diagnostics) was used with a probe against murine *Csrp3*, *Rcan1*, *Cryab*, *Nppb*, *Ankrd1* and *Tnnt2* mRNA as previously described^24^. Frozen sections (10 µm) were fixed in PBS containing 4% paraformaldehyde for 5 minutes at 19–22 °C, dehydrated by serial immersion in 50%, 70% and 100% ethanol for 5 minutes at room temperature and treated with protease for 30 minutes at room temperature. The probe was then hybridized for 2 hours at 40 °C, followed by RNAscope amplification. Samples were co-stained with DAPI to detect nuclei and wheat germ agglutinin (WGA) to detect cell membrane. Images were obtained using ×20 or ×63 objective on an LSM880 confocal microscope (Carl Zeiss). For image analysis, the HALO FISH-IF version 2.0 (Indica Labs) was applied to ×63 images to automatically detect mRNA spots and WGA signal as the cell border. To quantify the density of *Csrp3* mRNA molecules per cell, we measured the number of mRNA spots within each cardiomyocyte surrounded by WGA staining. Significance was determined by a one-way ANOVA with Bonferroniʼs multiple comparison test using GraphPad Prism 7.0e. Using the remained tissue section of the hearts, we also performed Masson’s trichrome staining as previously described^50^. All RNA in situ hybridizations were independently repeated two times to confirm that the similar results were obtained.

### Tissue histology

For histological analysis, mice were anesthetized by isoflurane inhalation and sacrificed by cervical dislocation. The chest was opened, and the heart was flushed with cold PBS via cardiac apical insertion of a 25-gauge needle. The right atrium was cut to allow drainage of blood from the heart, and the mice were briefly perfused with cold fixative (4% paraformaldehyde in PBS) through the apex of the heart. Tissues were excised, flushed with fixative, incubated in fixative for 12 hours at 4 °C with gentle rotation and finally embedded in paraffin. Paraffin-embedded heart tissues were sectioned into 4-μm slices using an SM2010 R Sliding Microtome (Leica Biosystems), and sections were stained using Picrosirius Red/Fast Green dyes. Bright-field histology images were taken using a ×10 objective on a BZ-X700 microscope (Keyence). All Picrosirius Red/Fast Green stainings were independently repeated three times to confirm that the similar results were obtained.

### qRT–PCR analysis

For qRT–PCR of *Csrp3*, multiple organs, such as heart, lung, liver, kidney, spleen, brain, intestine and skeletal muscle, were collected from mice after systemic perfusion with cold PBS. Total RNA was isolated from these tissues using TRIzol reagent (15596026, Thermo Fisher Scientific). After its purity was confirmed using the 260/280-nm absorbance (>1.8), single-stranded cDNA was synthesized using the High-Capacity cDNA Reverse Transcription Kit (4374966, Thermo Fisher Scientific) from 1 μg of RNA, following the manufacturer’s instructions. mRNA expression was evaluated by qRT–PCR using a CFX96 Real-Time PCR Detection System, and the relative expression levels of the target genes were normalized to the expression of an internal control gene, using the comparative Ct method. The result is shown as a bar graph, which was made using GraphPad Prism 7.0e. The following primer sets were used for qRT–PCR:

*Rps18* mRNA forward, CTTAGAGGGACAAGTGGCG

*Rps18* mRNA reverse, ACGCTGAGCCAGTCAGTGTA

*Csrp3* mRNA forward, TGAGAAGGTCATGGGAGGTG

*Csrp3* mRNA reverse, CTTGCTGTGTAAGCCCTCCA

### Reporting summary

Further information on research design is available in the Nature Research Reporting Summary linked to this article.

### Supplementary information

Reporting Summary

Supplementary Tables 1–7 Supplementary Table 1 The sequencing and alignment metrics produced by Cell Ranger. Supplementary Table 2 Top 150 genes or all genes (if fewer than 150) of positively differentially expressed genes (DEGs) of each cluster ranked by avg_log_2_FC in snRNA-seq. DEGs were defined as p_val_adj < 0.05 and avg_log_2_FC > 0.25. p_val_adj, *P* value adjusted for multiple testing based on Bonferroniʼs correction; avg log_2_FC, average log_2_ fold change between cluster of interest and all other clusters; Pct. 1, percentage of cells in the cluster with detectable marker expression; Pct. 2, percentage of cells in all other clusters with detectable marker expression. Supplementary Table 3 The sequencing and alignment metrics produced by Space Ranger. Supplementary Table 4 Top 100 genes or all genes (if fewer than 100) of positively differentially expressed genes (DEGs) of each cluster ranked by avg_log_2_FC in spatial transcriptomics. DEGs were difined as p_val_adj < 0.05 and avg_log_2_FC > 0.25. p_val_adj, *P* value adjusted for multiple testing based on Bonferroniʼs correction; avg log_2_FC, average log_2_ fold change between cluster of interest and all other clusters; Pct. 1, percentage of cells in the cluster with detectable marker expression; Pct. 2, percentage of cells in all other clusters with detectable marker expression. Supplementary Table 5 Gene lists of each gene module from WGCNA of spatial transcriptome. Supplementary Table 6 Table of the module membership as the correlation of the module eignegene and the gene expression profile from WGCNA of spatial transcriptome. Supplementary Table 7 Gene lists of each gene module from WGCNA of spatial transcriptome, including *Csrp3* knockdown and overexpression mice.

### Source data

Source Data Fig. 1 Top 150 genes or all genes (if fewer than 150) of positively differentially expressed genes (DEGs) of each cluster ranked by avg_log_2_FC in snRNA-seq. DEGs were defined as p_val_adj < 0.05 and avg_log_2_FC > 0.25. p_val_adj, *P* value adjusted for multiple testing based on Bonferroniʼs correction; avg log_2_FC, average log_2_ fold change between cluster of interest and all other clusters; Pct. 1, percentage of cells in the cluster with detectable marker expression; Pct. 2, percentage of cells in all other clusters with detectable marker expression.

Source Data Fig. 2 Top 100 genes or all genes (if fewer than 100) of positively differentially expressed genes (DEGs) of each cluster ranked by avg_log_2_FC in spatial transcriptomics. DEGs were defined as p_val_adj < 0.05 and avg_log_2_FC > 0.25. p_val_adj, *P* value adjusted for multiple testing based on Bonferroniʼs correction; avg log_2_FC, average log_2_ fold change between cluster of interest and all other clusters; Pct. 1, percentage of cells in the cluster with detectable marker expression; Pct. 2, percentage of cells in all other clusters with detectable marker expression.

Source Data Fig. 3 Table of the module membership as the correlation of the module eignegene and the gene expression profile from WGCNA of spatial transcriptome.

Source Data Fig. 3 Gene lists of each gene module from WGCNA of spatial transcriptome.

Source Data Fig. 4 Data of echocardiographic analysis. Gene lists of each gene module from WGCNA of spatial transcriptome, including Csrp3 knockdown and overexpression mice.

Source Data Extended Data Fig. 4 Pearson correlation coefficient with Csrp3 expression of each gene from single-cardiomyocyte RNA-seq.

## Data Availability

The sequencing and alignment metrics of snRNA-seq and Visium are provided as Extended Data tables. Single-cardiomyocyte, scRNA-seq and spatial transcriptomic data have been deposited in GSE176092 (https://www.ncbi.nlm.nih.gov/geo/query/acc.cgi?acc=GSE176092).

## Code availability

Scripts for processing the transcriptome data are available on GitHub (https://github.com/firstheart123/spatiotemporal_heart).
