# Supplementary material for: Spatiotemporal transcriptome analysis reveals critical roles for mechano-sensing genes at the border zone in remodeling after myocardial infarction
Source: Nat Cardiovasc Res. 2022 Nov 17;1(11):1072–83. doi: 10.1038/s44161-022-00140-7 (PMC11358009; doi:10.1038/s44161-022-00140-7)
Supplement: Supplementary file 1 — Reporting Summary [file 44161_2022_140_MOESM1_ESM.pdf]

nature research

Corresponding author(s): Issei Komuro

Last updated by author(s): Jul 25, 2022

Reporting Summary

Nature Research wishes to improve the reproducibility of the work that we publish. This form provides structure for consistency and transparency in reporting. For further information on Nature Research policies, see our Editorial Policies and the Editorial Policy Checklist.

Statistics

For all statistical analyses, confirm that the following items are present in the figure legend, table legend, main text, or Methods section.

n/a	Confirmed	
☐	☒	The exact sample size (*n*) for each experimental group/condition, given as a discrete number and unit of measurement
☐	☒	A statement on whether measurements were taken from distinct samples or whether the same sample was measured repeatedly
☐	☒	The statistical test(s) used AND whether they are one- or two-sided<br>*Only common tests should be described solely by name; describe more complex techniques in the Methods section.*
☒	☐	A description of all covariates tested
☒	☐	A description of any assumptions or corrections, such as tests of normality and adjustment for multiple comparisons
☐	☒	A full description of the statistical parameters including central tendency (e.g. means) or other basic estimates (e.g. regression coefficient) AND variation (e.g. standard deviation) or associated estimates of uncertainty (e.g. confidence intervals)
☐	☒	For null hypothesis testing, the test statistic (e.g. *F*, *t*, *r*) with confidence intervals, effect sizes, degrees of freedom and *P* value noted<br>*Give P values as exact values whenever suitable.*
☒	☐	For Bayesian analysis, information on the choice of priors and Markov chain Monte Carlo settings
☒	☐	For hierarchical and complex designs, identification of the appropriate level for tests and full reporting of outcomes
☒	☐	Estimates of effect sizes (e.g. Cohen's *d*, Pearson's *r*), indicating how they were calculated

Our web collection on statistics for biologists contains articles on many of the points above.

Software and code

Policy information about availability of computer code

Data collection	Microscope: BZ-X700 microscope (Keyence). Echocardiography: Vevo 2100 imaging system (Visualsonics, Inc.). qPCR: CFX96 Real-Time PCR Detection System (Bio-Rad). DNA electrophoresis: LabChip GX (Perkin Elmer), TapeStation 2200 (Agilent Technologies). Western blot: LAS 4000 analyser (GE Healthcare).
Data analysis	Single-cell, single-nucleus and spatial RNA-seq analysis: R (v4.1.2), fastp (v0.21.0), STAR (v2.7.8), featureCounts (v2.0.2), Seurat (v4.1.1), WGCNA (v1.6.9), Cytoscape (v3.8.0), Cell Ranger (v6.1.1), Space Ranger (v1.2.1), CARD (v1.0), Metascape (http://metascape.org/gp/index.html). Imaging analysis: BZ Analyzer software (v1.1.1.8, Keyence). Statistical analysis: GraphPad Prism 7.0e

For manuscripts utilizing custom algorithms or software that are central to the research but not yet described in published literature, software must be made available to editors and reviewers. We strongly encourage code deposition in a community repository (e.g. GitHub). See the Nature Research guidelines for submitting code & software for further information.

Data

Policy information about availability of data

All manuscripts must include a data availability statement. This statement should provide the following information, where applicable:

- Accession codes, unique identifiers, or web links for publicly available datasets
- A list of figures that have associated raw data
- A description of any restrictions on data availability

The sequencing and alignment metrics of single-nucleus RNA-seq and Visium are provided as supplementary tables. Single-cardiomyocyte, single-cell RNA sequencing, and spatial transcriptomic data have been deposited in GSE176092 (https://www.ncbi.nlm.nih.gov/geo/query/acc.cgi?acc=GSE176092).

Field-specific reporting

Please select the one below that is the best fit for your research. If you are not sure, read the appropriate sections before making your selection.

☒ Life sciences ☐ Behavioural & social sciences ☐ Ecological, evolutionary & environmental sciences

For a reference copy of the document with all sections, see nature.com/documents/nr-reporting-summary-flat.pdf

Life sciences study design

All studies must disclose on these points even when the disclosure is negative.

Sample size	No statistical methods were used to predetermine sample size estimates (39). Sample size was determined based on the experimental results that we obtained from preliminary experiments and published papers. In vivo studies, we used standard sample sizes reported in the literature previously in mouse studies. The numbers of performed experiments were indicated in each figure legend.
Data exclusions	Before starting in vivo experiments, we have established the criteria that mice that failed to develop MI or died within one week after the operation were excluded from the analysis.
Replication	Major experiments were performed with an appropriate sample size to obtain statistical significance. To keep the number of animals used in experiments as low as possible, animal experiments related with AAV9 injection were performed with limited sample size. To make sure that the experiments could be reproduced, more than one person was often involved in major experiments (e.g. operation of mice, injection of AAV9, echocardiographic analysis, RNA-seq analysis, and RNA in situ hybridization).<br>The numbers of replication are follows. snRNA-seq, n = 2; spatial transcriptome, n = 3; RNA in situ hybridization, n = 2.
Randomization	In vivo experiments, mice were randomly allocated to each group.
Blinding	Operation and echocardiographic analysis, snRNA-seq and spatial trascriptome analyses of mice were obtained by independent operators who were blinded to condition of mice.

Reporting for specific materials, systems and methods

We require information from authors about some types of materials, experimental systems and methods used in many studies. Here, indicate whether each material, system or method listed is relevant to your study. If you are not sure if a list item applies to your research, read the appropriate section before selecting a response.

Materials & experimental systems

n/a	Involved in the study	
☒	☐	Antibodies
☒	☐	Eukaryotic cell lines
☒	☐	Palaeontology and archaeology
☐	☒	Animals and other organisms
☒	☐	Human research participants
☒	☐	Clinical data
☒	☐	Dual use research of concern

Methods

n/a	Involved in the study	
☒	☐	ChIP-seq
☒	☐	Flow cytometry
☒	☐	MRI-based neuroimaging

Animals and other organisms

Policy information about studies involving animals; ARRIVE guidelines recommended for reporting animal research

Laboratory animals	Mice were housed in a specific pathogen-free facility with a 12-h light/12-h dark cycle. Ambient room temperature was regulated at 73±5 °F and humidity was controlled at 50±10 %. C57BL/6 were purchased from CLEA JAPAN. Age of mice operated and used for experiments were 9-11 week old male mice.
Wild animals	The study did not involve wild animals.
Field-collected samples	The study did not involve field collected samples.
Ethics oversight	The University of Tokyo Ethics Committee for Animal Experiments

Note that full information on the approval of the study protocol must also be provided in the manuscript.
